# Supplementary material for: Induction of DISE in ovarian cancer cells in vivo
Source: Oncotarget. 2017 Oct 4;8(49):84643–58. doi: 10.18632/oncotarget.21471 (PMC5689563; doi:10.18632/oncotarget.21471)
Supplement: Supplementary file 1 [file oncotarget-08-84643-s001.pdf]

Induction of DISE in ovarian cancer cells *in vivo*

SUPPLEMENTARY MATERIALS

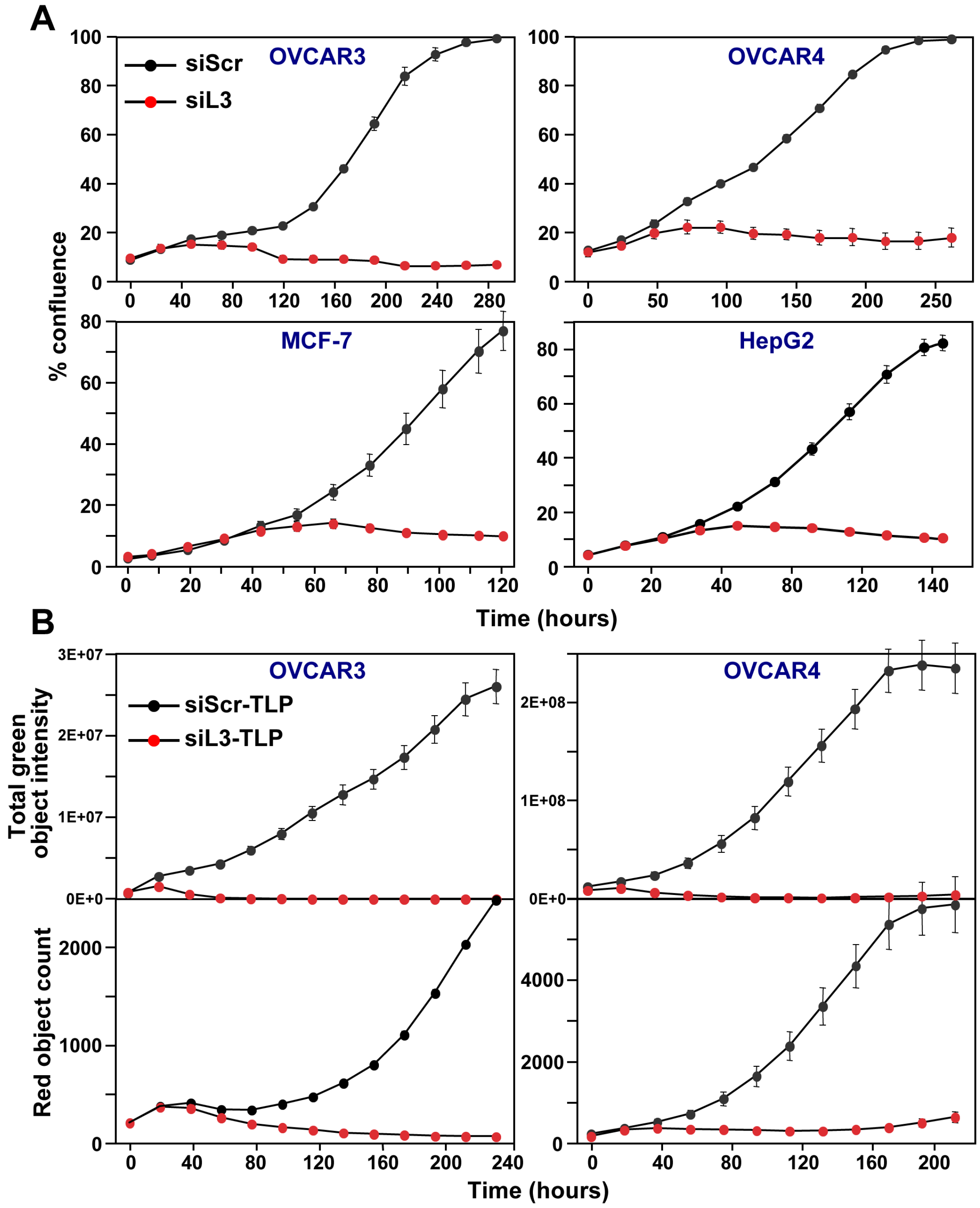

Supplementary Figure 1: Induction of DISE by siL3 in different cancer cell lines. A: Confluency over time of four cancer cell lines transfected with 10 nM (OVCAR3, OVCAR4 or HepG2) or 25 nM (MCF-7) siScr or siL3. **B**: *Top:* Total green intensity or *bottom*: confluency over time of OVCAR3 Venus siL3 or OVCAR4 Venus siL3 cells treated with 30 nM of either siScr-TLP or siL3-TLP.

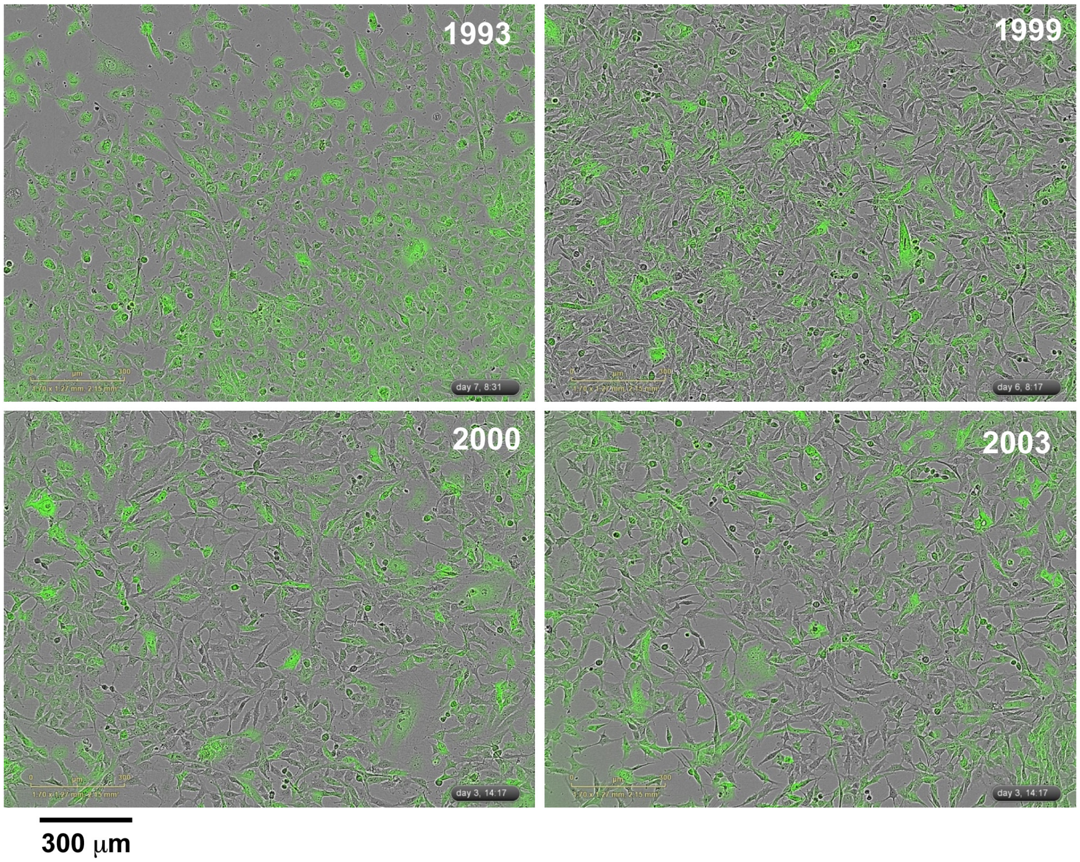

Supplementary Figure 2: Combined phase contrast and green fluorescence of cells isolated from the four tumors used in Figure 2C.

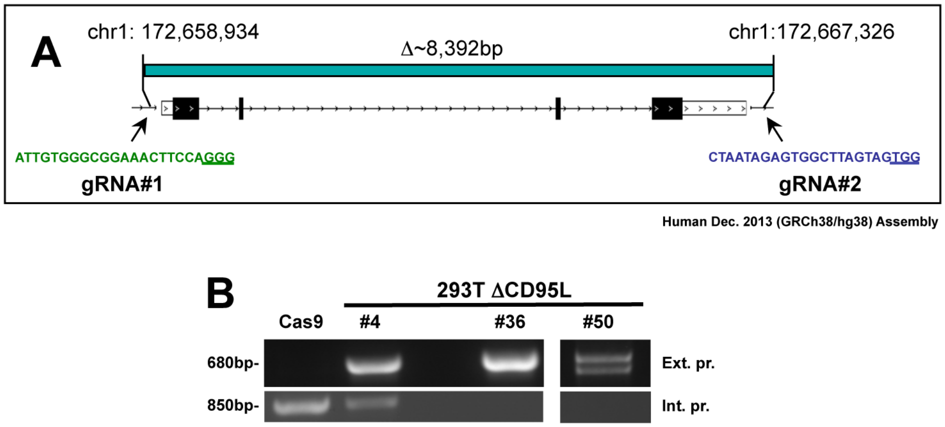

Supplementary Figure 3: Generation of homozygous CD95L deletion in 293T cells. **A**: Schematic of the genomic locations and sequences of the gRNAs used to excise the entire CD95L gene. Blue indicates the gRNA targeting the antisense strand. Green indicates the gRNA targeting the sense strand. PAM site is underlined. **B**: PCR with flanking (*top panels*) and internal (*bottom panels*) primers was used to confirm the absence of CD95L in 293T clones. Cells infected with Cas9 only (Cas9) are wild-type, clone #4 is heterozygous, and clones #36 and #50 carry homozygous deletions.

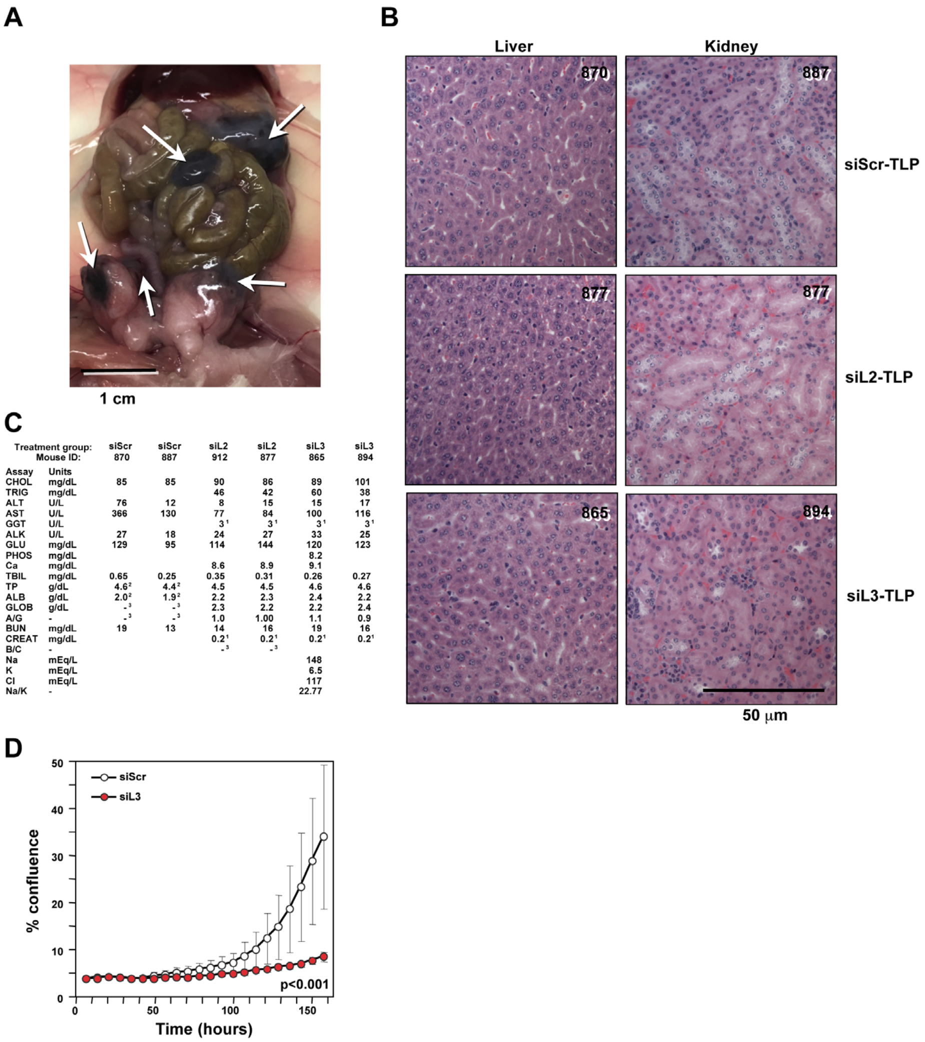

Treatment group:		siScr	siScr	siL2	siL2	siL3	siL3
Mouse ID:		870	887	912	877	865	894
Assay	Units						
CHOL	mg/dL	85	85	90	86	89	101
TRIG	mg/dL			46	42	60	38
ALT	U/L	76	12	8	15	15	17
AST	U/L	366	130	77	84	100	116
GGT	U/L			3[1]	3[1]	3[1]	3[1]
ALK	U/L	27	18	24	27	33	25
GLU	mg/dL	129	95	114	144	120	123
PHOS	mg/dL					8.2	
Ca	mg/dL			8.6	8.9	9.1	
TBIL	mg/dL	0.65	0.25	0.35	0.31	0.26	0.27
TP	g/dL	4.6[2]	4.4[2]	4.5	4.5	4.6	4.6
ALB	g/dL	2.0[2]	1.9[2]	2.2	2.3	2.4	2.2
GLOB	g/dL	-[3]	-[3]	2.3	2.2	2.2	2.4
A/G	-	-[3]	-[3]	1.0	1.00	1.1	0.9
BUN	mg/dL	19	13	14	16	19	16
CREAT	mg/dL			0.2[1]	0.2[1]	0.2[1]	0.2[1]
B/C	-			-[3]	-[3]		
Na	mEq/L					148	
K	mEq/L					6.5	
Cl	mEq/L					117	
Na/K	-					22.77	

Supplementary Figure 4: No toxicity of siL3-TLP in NGS mice. A: Representative image of mouse peritoneum with HeyA8 tumor stained grey caused by the gold containing TLPs (arrows). **B**: Representative H&E staining of liver and kidney sections from mice of the three treatment groups. **C**: Serum analysis of two mice per treatment group. 1 = Sample assay value is less than the dynamic range. For most assays, the dynamic range low limit is reported. 2 = Sample was diluted for testing. Assay value for sample was below dynamic range, but results have been corrected for dilution. 3 = Assay is a calculated value. Either or both assay values used in the calculation were below the dynamic range of the assay, therefore no result is reported. **D**: Induction of DISE in ID8-Venus-mFasL cells after transfection with 25 nM siL3. As a control cells were transfected with siScr. ANOVA was performed for pairwise comparison of change in confluence over time between siScr and siL3 treated cells.

For Supplementary Movies see in Supplementary Files
Supplementary Movie 1: HeyA8-pTIP-shL3 cells.
Supplementary Movie 2: HeyA8-pTIP-shL3 cells plus Dox.
Supplementary Movie 3: HeyA8-pTIP-shL3 H5 clone growing in the presence of Dox. Resistant cells are growing into the field from the right.
Supplementary Movie 4: HeyA8-pTIP-shScr control cells growing in the presence of Dox.

Supplementary Movie 5: HeyA8 Venus siL3WT cells treated with siL3MUT.

Supplementary Movie 6: HeyA8 Venus siL3WT treated with siL3.

Supplementary Movie 7: HeyA8 Venus siL3MUT treated with siL3MUT.

Supplementary Movie 8: HeyA8 Venus siL3MUT treated with siL3.
